# Minorities with lupus nephritis and medications: a study of facilitators to medication decision-making

**DOI:** 10.1186/s13075-015-0883-z

**Published:** 2015-12-17

**Authors:** Jasvinder A. Singh, Haiyan Qu, Jinoos Yazdany, Winn Chatham, Richard Shewchuk

**Affiliations:** Medicine Service, Birmingham VA Medical Center, 510, 20th street South, FOT 805B, Birmingham, AL USA; Department of Medicine at School of Medicine, and Division of Epidemiology at School of Public Health, University of Alabama, 1720 Second Ave. South, Birmingham, AL 35294-0022 USA; Department of Orthopedic Surgery, Mayo Clinic College of Medicine, 200 1st St SW, Rochester, MN 55905 USA; Department of Health Services Administration at School of Health Professions, University of Alabama at Birmingham, 1705 University Blvd, Birmingham, AL 35233 USA; Department of MedicineUniversity of California at San Francisco, 505 Parnassus Ave, San Francisco, CA 94143 USA

**Keywords:** Lupus, Systemic lupus erythematosus, Hispanic, African-American, Minorities, Race, Facilitators, Challenges, Immunosuppressive medication, Adherence

## Abstract

**Background:**

Medication decision-making poses a challenge for a significant proportion of patients. This is an even more challenging for patients who have complex, rare, immune conditions that affect them at a young age and are associated with the use of life-long treatment, perceived by some as having significant risk of side effects and toxicity.

**Introduction:**

The aim of our study was to examine the perspectives of women with lupus nephritis on facilitators to medication decision-making.

**Methods:**

We used the nominal group technique (NGT), a structured formative process to elicit patient perspectives. An NGT expert moderated eight patient group meetings. Participants (n = 52) responded to the question “What sorts of things make it easier for people to decide to take the medicines that doctors prescribe for treating their lupus kidney disease?” Patients nominated, discussed, and prioritized facilitators to medication decisional processes.

**Results:**

Fifty-two women with lupus nephritis participated in eight NGT meetings (27 African-American, 13 Hispanic, and 12 Caucasian). Average age was 40.6 years (standard deviation (SD) = 13.3), and disease duration was 11.8 years (SD = 8.3); 36.5 % obtained at least a college education, and 55.8 % had difficulty in reading health materials. Patients generated 280 decision-making facilitators (range of 26 to 42 per panel). Of these, 102 (36 %) facilitators were perceived by patients as having relatively more influence in decision-making processes than others. Prioritized facilitators included effective patient-physician communication regarding benefits/harms, patient desire to live a normal life and improve quality of life, concern for their dependents, experiencing benefits and few/infrequent/no harms with lupus medications, and their affordability. Relative to African-Americans, Caucasian and Hispanic patients endorsed a smaller percentage of facilitators as influential. Level of agreement with which patients within panels independently agreed in their selections of the three most influential facilitators ranged from 33 % to 60 %.

**Conclusions:**

We identified facilitators to lupus medication decision-making. This information will be used to populate a decision aid for lupus nephritis.

**Electronic supplementary material:**

The online version of this article (doi:10.1186/s13075-015-0883-z) contains supplementary material, which is available to authorized users.

## Background

Lupus nephritis, if not treated promptly with appropriate medications, is a common manifestation of lupus that can lead to end-stage renal disease and dialysis [1]. Many patients with lupus do not take their medications regularly [2]. Although the precise reasons for this are not known, qualitative research has focused mainly on disease experience [3–8] or long-term need for these medications [9] and only in some cases on barriers to medication intake [9–12] as important contributors to this difficult clinical problem. A systematic review of studies focused on lupus medications identified treatment adherence as one of the five main themes in the management of patients with lupus [13]. In particular, effective communication by clinicians promoted a sense of trust and respect among patients with lupus, and “medication adherence was their way of demonstrating their appreciation”.

On the other hand, it is not known what factors help patients in making a decision to start taking their lupus medications. This is a large gap in the literature. Medication decision-making poses a challenge for a significant proportion of patients [14]. Therefore, we undertook the current study. Unlike investigators in previous qualitative work in the area of medication adherence, we used the nominal group technique (NGT) as a more structured approach to elicit both qualitative (ideas) and quantitative (ranking) data from patients [15]. Our objective was to identify a comprehensive array of patient-reported facilitators and the relative benefit each was perceived to have in the medication decision-making process for women with lupus nephritis. We oversampled racial/ethnic minorities in our study, given that the severity of lupus symptoms is higher and outcomes are worse for minorities with lupus [16, 17]. Our research was guided by a single question aimed at identifying factors that facilitated decisional processes involving medications for treating lupus nephritis: “What sorts of things make it easier for people to decide to take the medicines that doctors prescribe for treating their lupus kidney disease?”

## Methods

### Study cohort

We recruited patients from the lupus clinics at the University of Alabama at Birmingham (UAB) and the University of California at San Francisco (UCSF). All patients met American College of Rheumatology classification criteria for systemic lupus erythematosus and had a clinical diagnosis of lupus nephritis (based on renal biopsy or laboratory tests or both).

We convened eight NGT meetings including lupus nephritis patients who had received treatment and were following at UAB or UCSF lupus clinics. An expert NGT researcher (RS) conducted and moderated all NGT meetings in English between February and April 2014. The institutional review boards at UAB and UCSF approved this study. All patients provided written informed consent.

### Nominal group technique

The NGT meeting is a facilitated data collection activity structured to promote even and equal subject participation by minimizing the loss of information. Evidence shows that the NGT, when used correctly, elicits a greater volume of novel and higher-quality responses in response to a carefully articulated question than the less structured group data collection approaches such as focus groups and brainstorming [18, 19]. Moreover, by using the verbatim responses that are concisely documented on a flip chart as participants present them to the group, the NGT eliminates a potential source of investigator-induced interpretive bias resulting from transcribing and coding audio or video recordings.

The purpose of NGT meetings was to tap into patients’ unique insights, knowledge, and lived experiences to identify different factors that facilitated their decision-making process involving prescribed lupus medications. The NGT leader (RS) along with a team member (HQ) started the sessions with a brief explanation of the purpose and the NGT process. Patients then worked independently for about five minutes to develop their own lists of brief statements/phrases in response to the NGT question.

Patients were encouraged to think broadly about the types of things that enhanced the likelihood of deciding to take the medications prescribed for their condition. This ensured that each panel generated a wide array of responses. After five minutes of working on their own, patients were invited to present their responses to the group. To promote open disclosure, increase response volume, and ensure that all patients had an equal opportunity to present responses, we used a “round-robin” participation format. This format involved having each patient, in turn, articulate a single response without providing any rationale, justification, or explanation for their response and without discussion or debate from other members in the group. All responses were immediately recorded verbatim on a flip chart to help participants recollect previously nominated responses. We continued until no further responses could be generated. All responses were then discussed in a non-evaluative fashion to ensure that they were understood from a common perspective and potentially to obtain additional insights [15].

Patients were asked to silently review the full list of responses generated during the meeting and to independently select three facilitators that they perceived as the most influential in their decision-making regarding lupus nephritis medication. Patients recorded their selected responses on index cards and prioritized the influence each of their selections from 1 (least influential) to 3 (most influential). The votes reflecting these priorities were tabulated across patients in each NGT panel to determine the perceived relative influence of medication decision-making facilitators and the level of agreement among patients regarding these perceptions.

A brief questionnaire was administered at the conclusion of each NGT meeting to obtain basic demographic data, education level, disease duration and whether the patient needed assistance in reading materials. Data from this questionnaire were analyzed at the group level and not linked with individual responses generated during the NGT meetings.

## Results

Fifty-two patients with lupus nephritis participated in eight NGT meetings. Mean age was 40.6 years (standard deviation (SD) = 13.3), and average disease duration was 11.8 years (SD = 8.3); 36.5 % had obtained at least a college degree, and 55.8 % indicated a need for some help (from a family member, friend, and hospital or clinic staff) in reading health materials (Table 1). Twenty-seven were African-American (four nominal groups), 13 were Hispanic (two nominal groups), and 12 were Caucasian (two nominal groups).

**Table 1:** Participant characteristics by nominal group technique meeting panel (n = 52)

NGT group (N)	Age	Age of diagnosis	Education		Need help in reading health materials		Disease activity in past 3 months^a^
			Below college	College or above	No	Yes	
	Mean (SD)	Mean (SD)	N (%)	N (%)	N (%)	N (%)	Mean (SD)
AA1 (9)	36.9 (13.1)	26.4 (12.4)	7 (77.8)	2 (22.2)	5 (55.6)	4 (44.6)	6.1 (2.7)
AA2 (7)	49.1 (4.8)	36.4 (6.3)	6 (85.7)	1 (14.3)	4 (57.1)	3 (42.9)	4.7 (2.8)
AA3 (7)	38.1 (11.9)	28.9 (14.0)	3 (42.9)	4 (57.1)	4 (57.1)	3 (42.9)	5.0 (2.9)
AA4 (4)	42.5 (14.9)	36.1 (18.1)	2 (50.0)	2 (50.0)	1 (25.0)	3 (75.0)	7.3 (3.0)
CA1 (6)	47.3 (19.9)	32.8 (19.3)	5 (83.3)	1 (16.7)	5 (83.3)	1 (16.7)	5.0 (3.2)
CA2 (6)	45.7 (11.5)	32.4 (14.2)	2 (33.3)	4 (66.7)	1 (16.7)	5 (83.3)	3.3 (2.9)
HA1 (6)	31.7 (12.2)	21.2 (4.3)	5 (83.3)	1 (16.7)	5 (83.3)	1 (16.7)	4.0 (2.4)
HA2 (7)	35.4 (12.0)	18.3 (9.3)	4 (57.1)	3 (42.9)	4 (57.1)	3 (42.9)	4.0 (3.6)
Total (52)	40.6 (13.3)	28.5 (13.3)	34 (65.4)	18 (35.6)	29 (55.8)	23 (44.2)	4.9 (2.9)

*NGT* nominal group technique, *N* number, *SD* standard deviation, *AA* African American, *CA* Caucasian American, *HA* Hispanic American

^a^Disease activity in past 3 months was measured by using a patient self-reported 0–10 rating scale

Patients generated 280 decision-making facilitators (range from 26 to 42 facilitators per panel) (Table 2). Of these, 102 (36 %) facilitators were perceived by patients as having relatively more influence in their own decision-making processes (i.e., were responses selected from each panel’s generated list of responses and then assigned weighted votes) than responses reflecting other facilitators. Differences in the number of prioritized responses as a percentage of total generated responses were observed across the panels (range from 31 % to 52 %).

**Table 2:** Summary statistics for nominal group technique meetings (n = 52)

Group	# of participants (N)	# of responses	# of responses per participant	# of prioritized responses (R)	% of prioritized responses (%)	Rescaled agreement (%)^a^
AA1	9	31	3.4	16	51.6	45.8
AA2	7	35	5.0	14	40.0	38.9
AA3	7	37	5.3	13	35.1	44.4
AA4	4	26	6.5	8	30.8	44.4
CA1	6	34	5.7	11	32.3	46.7
CA2	6	38	6.3	14	36.8	26.7
HA1	6	38	6.3	13	34.2	33.3
HA2	7	42	6.0	13	30.9	44.4
Total	52	281	5.4	102	36.3	35.3

*AA* African American, *CA* Caucasian American, *HA* Hispanic American

^a^Rescaled agreement = (3 N − R)/ (3 N − 3) × 100, where N = number of participants, and R = number of prioritized responses

Relative to African-American patients, Caucasian and Hispanic patients tended to endorse a smaller percentage of facilitators as influential (African-American range from 41 %–54 % versus Caucasian 32 %–35 % and Hispanic 35 %–38 %). Rescaled values expressing the level of agreement or consistency with which patients within panels independently agreed in their selections of the three most influential decision-making facilitators ranged from 33 % for African-American panels (panels 3 and 4) and Hispanic panel 1 to 60 % for Caucasian panel 1 (Table 2).

We attempted to summarize results across the eight NGTs by race/ethnicity, but the prioritized responses were too different to allow this grouping. Therefore, facilitators are presented for each nominal group in the section below.

### NGT group results

**Fig. 1:**
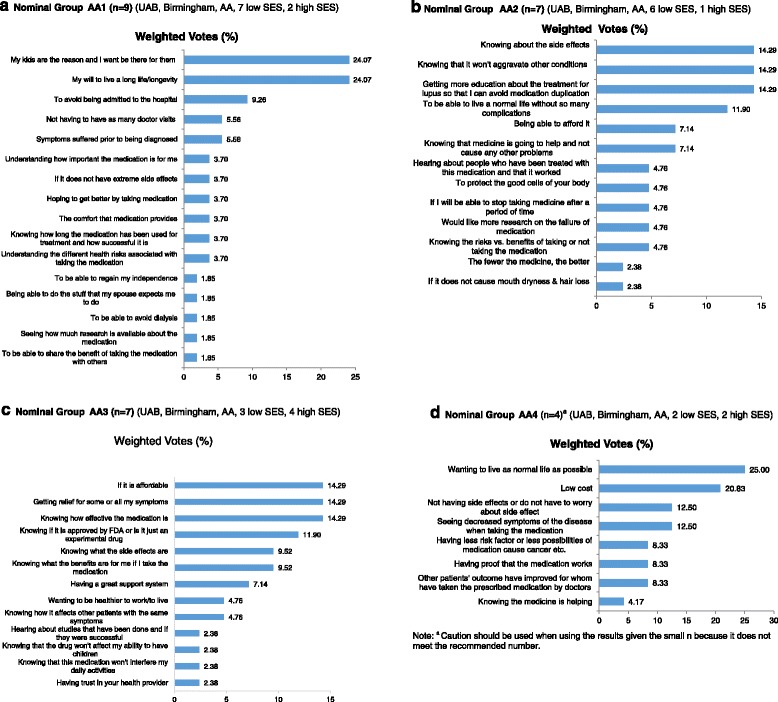
Prioritized facilitators to help patients make decisions about treatment choices in African-American patients in nominal groups 1 (**a**), 2 (**b**), 3 (**c**) and 4 (**d**). *AA* African- American, *SES* socioeconomic status, *UAB* University of Alabama at Birmingham

The first NGT meeting was conducted at the UAB site and involved nine African-American women patients who were between 27 and 66 years of age (mean = 36.9 and SD = 13.15). Most of the patients in this group (7 out of 9) reported their education level as less than a college degree. Patients in this group generated 31 responses reflecting potential facilitators of the medication decision-making process. As indicated by their independent assignment of votes (Fig. 1a; see Additional file 1 for more details), the patients who participated in this meeting collectively endorsed 16 facilitators as relatively more influential than others with respect to their own decision-making. The relative influence of each selected facilitator was then assessed in consideration of both the number of patients who endorsed it as “personally influential” and the weighted votes it was assigned in the prioritization exercise. The results from the first NGT meeting indicate that 4 of the 16 medication decision-making facilitators were endorsed as being influential by multiple patients and were assigned 34 (63 %) of the 54 available weighted votes (Fig. 1; see Additional file 1 for more details). Presented relative to the “group-level” perceived influence, the four facilitators generally concern (1) the desire to be present or available for one’s children (endorsed by 5 out of 9 patients; 24 % weighted votes), (2) the will to live a longer life (endorsed by 5 out of 9 patients; 24 % weighted votes), (3) avoiding hospitalization (endorsed by 2 out of 9 patients; 9 % weighted votes), and (4) reducing the need for doctor visits (endorsed by 3 out of 9 patients; 6 % weighted votes) (Fig. 1; see Additional file 1 for more details).

The second NGT meeting was conducted at UAB with seven African-American women patients who were on average 49.1 (SD = 4.8 ; range,  42 to 56) years of age. Six patients reported not having obtained a college degree, and one patient reported that she had obtained a college degree. During this meeting, patients generated 35 responses that reflected their thinking about medication decision-making facilitators. Acting independently, patients in this group subsequently endorsed 14 of the 35 facilitators as having relatively more influence than the facilitators that were not endorsed (Fig. 1b; see Additional file 2 for more details). Based on multiple endorsements and the assignment of weighted votes, patients in group 2 collectively perceived 6 of the 14 facilitators as having had the greatest influence on their personal medication decision-making processes. As a group, the six facilitators were assigned 29 (69.1 %) of the 42 available weighted votes (Fig. 1b; see Additional file 2 for more details). Patients in this group were evenly divided with respect to what they considered the most influential facilitator as indicated by the similar endorsement profile associated with three different facilitators. Specifically, two patients each endorsed and assigned 14 % of the available votes to facilitators concerning (1) having knowledge about medication side effects generally, (2) having knowledge about medication side effects and how it affects other conditions or organs, and (3) obtaining medication education as a way to avoid overlap or duplication with other medication regiments. At least two patients also endorsed the influence of facilitators concerning (4) the desire to live a more normal life (endorsed by 2 out of 7 patients; 12 % weighted votes), (5) medication affordability (endorsed by 3 out of 7 patients, 7 % weighted votes), and (6) assurance that the prescribed medication is likely to help without causing other problems (endorsed by 2 out of 7 patients; 7 % weighted votes).

A third NGT meeting was conducted at UAB with an additional seven African-American women who had a mean age of 38.1 (SD = 11.9 ; range,  20 to 52) years. Four of 7 patients reported that they have received a college degree or above. This group generated 37 facilitators and selected 13 facilitators as having more influence on their own medication decision-making processes than the other 24 facilitators. Based on multiple endorsements and the weighted votes criteria, patients in this meeting identified six facilitators that accounted for almost 74 % of the weighted votes (31 out of 42) available for prioritizing facilitator influence (Fig. 1c; see Additional file 3 for more details). The six facilitators concerned (1) affordability (endorsed by 4 out of 7 patients; 14 % weighted votes), (2) the potential of the medication to provide symptom relief (endorsed by 2 of 7 patients, 14 % weighted votes), (3) having knowledge of medication effectiveness (endorsed by 2 out of 7 patients; 14 % weight votes), (4) assurance that the medication is “approved” and not experimental (endorsed by 2 out of 7 patients; 12 % weighted votes), (5) having knowledge of side effects (endorsed by 2 out of 7 patients; 10 % weighted votes), and (6) understanding what the benefits of the medication will be (2 out of 7 patient endorsements; 10 % weighted votes) (Fig. 1c; see Additional file 3 for more details).

A fourth NGT meeting at UAB involved four African-American patients who had a mean age of 42.5 years (SD = 14.9 ; range,  26 to 57 years). Two patients in this group reported having obtained at least a college degree, and two had not. The patients who participated in this meeting generated a list of 26 responses representing their views about medication decision-making facilitators. From this list, they independently endorsed eight facilitators as being relatively more influential in their own medication decisions (Fig. 1d; see Additional file 4 for more details). Three facilitators were endorsed as influential by at least two patients. These accounted for 59 % of the available weighted votes and concerned (1) the desire to live a normal life (endorsed by 2 out of 4 patients; 25 % weighted votes), (2) medication affordability or low cost (endorsed by 3 out of 4 patients; 21 % weighted votes), and (3) not having to worry about side effects (endorsed by 2 out of 4 patients; 13 % weighted votes) (Fig. 1d; see Additional file 4 for more details).

**Fig. 2:**
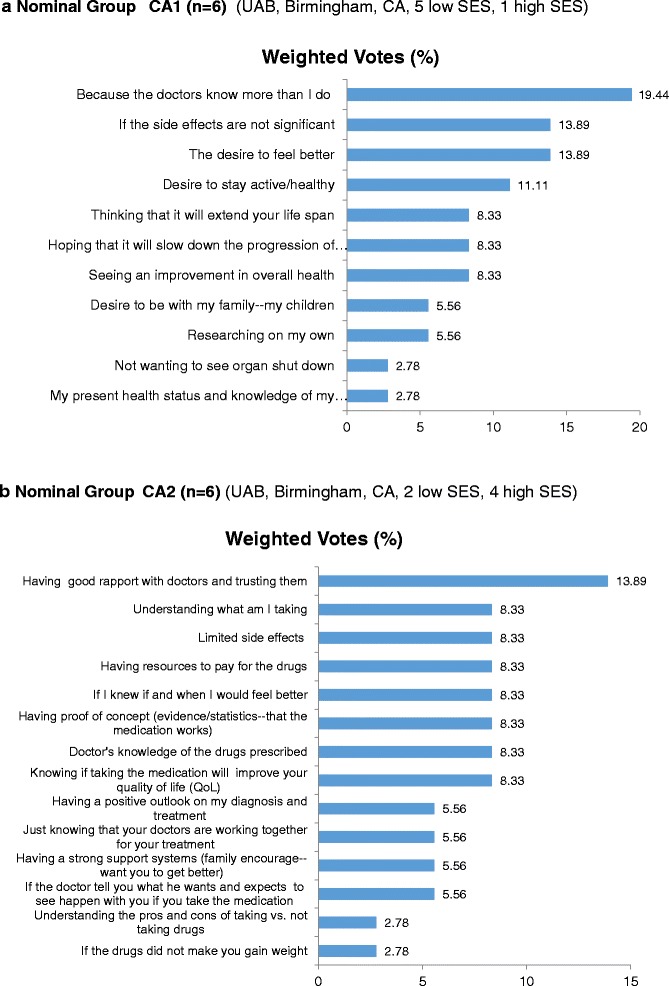
Prioritized facilitators to help patients make decisions about treatment choices in Caucasian patients in nominal groups 1 (**a**) and 2 (**b**). *CA*, Caucasian, *SES* socioeconomic status, *UAB* University of Alabama at Birmingham

A fifth NGT meeting involved six Caucasian women who were patients at UAB. The patients in this group had a mean age of 47.3 years (SD = 19.9 ; range,  21 to 72). One of the patients reported having obtained a college degree, and five patients reported having less education. As a group, they generated a list of 34 facilitators and independently selected 11 facilitators from this list as having more influence on their own medication decision-making than other facilitators (Fig. 2a; see Additional file 5 for more details). Five facilitators were each endorsed as influential by at least two patients and were assigned almost 67 % of the available weighted votes. The facilitators concerned (1) the belief that their doctors were more knowledgeable than they were themselves (3 out of 6 patient endorsements; 19 % weighted votes), (2) lack of significant medication side effects (endorsed by 3 out of 6 patients; 14 % weighted votes), (3) having the desire to feel better (endorsed by 2 out of 6 patients; 14 % weighted votes), (4) having the desire to stay active (endorsed by 2 out of 6 patients; 11 % weighted votes), and (5) belief that the medication will increase longevity (endorsed by 2 out of 6 patient endorsements; 8 % weighted votes) (Fig. 2a; see Additional file 5 for more details).

The sixth and final NGT meeting conducted at UAB involved six Caucasian women patients who had a mean age of 45.7 years (SD = 11.5 ; range,  24 to 74). Four patients in this group indicated that they obtained at least a college degree. Patients in this group generated 38 responses reflecting their views of potential medication decision-making facilitators and subsequently endorsed 14 of these as relatively more influential than others (Fig. 2b; see Additional file 6 for more details). At least two patients from this group assigned one of their three weighted votes to each of four facilitators, which accounted for about 36 % of the weighted votes available for prioritizing facilitator influence. These facilitators were (1) having trust and rapport with physician (endorsed by 2 out of 6 patients; 14 % weighted votes), (2) having an understanding of medication (endorsed by 2 out of 6 patients; 8 % weighted votes), (3) limited side effects (endorsed by 2 out of 6 patients, 8 % weighted votes), and (4) keeping a positive outlook about diagnosis and treatment (endorsed by 2 out of 6 patients; 6 % weighted votes) (Fig. 2b; see Additional file 6 for more details).

**Fig. 3:**
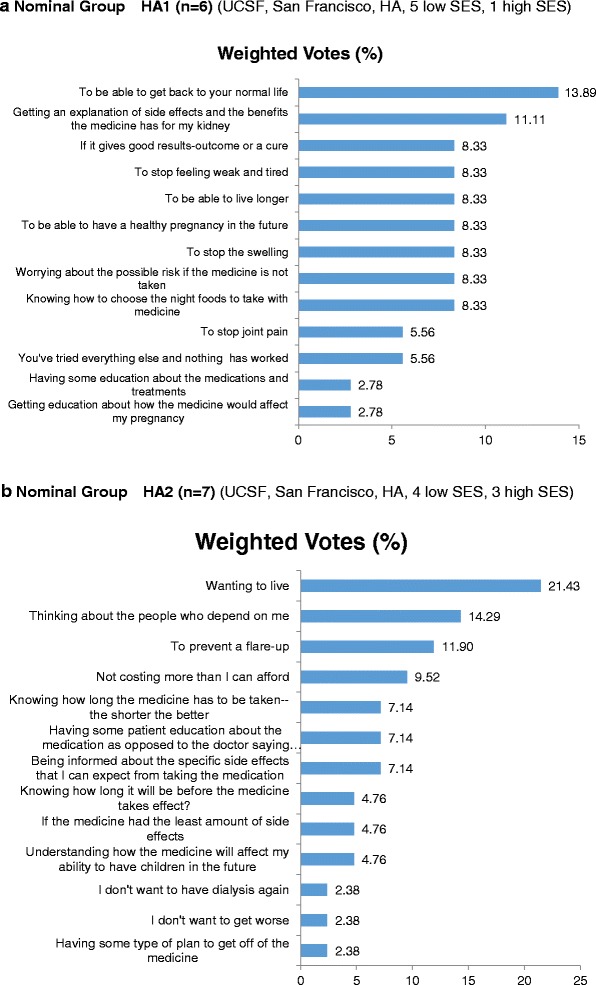
Prioritized facilitators to help patients make decisions about treatment choices in Hispanic patients in nominal groups 1 (**a**) and 2 (**b**). *HA* Hispanic, *SES* socioeconomic status, *UAB* University of Alabama at Birmingham

A seventh NGT meeting was conducted at UCSF with a group of six Hispanic American women. The patients in this group had a mean age of 31.7 years (SD = 12.2 ; range,  19 to 51), and 5 out of 6 patients reported that they did not have a college degree. This group generated 38 responses describing potential facilitators of medication decision-making. From this total, they selected 13 facilitators as being relatively more influential than others in terms of their own medication decision-making processes. At least two patients endorsed each of four facilitators as influential and assigned almost 42 % of available weighted votes to them (Fig. 3a; see Additional file 7 for more details). The four facilitators concerned (1) motivation to return to a normal life (endorsed by 3 out of 6 patients; 14 % weighted votes), (2) receiving explanations of medication benefits and side effects (endorsed by 2 of 6 patients; 11 % weighted votes), (3) having assurance of results (cure) (endorsed by 2 of 6 patients; 8 % weighted votes), and (4) to overcome fatigue and weakness (endorsed by 2 of 6 patients; 8 % weighted votes). It should be noted that the six patients who participated in this meeting each endorsed a different facilitator as most influential in their own decision-making process (Fig. 3a; see Additional file 7 for more details).

The eighth and final NGT was also conducted at UCSF and involved seven Hispanic American women who were on average 35.4 years of age (SD = 12.0 ; range,  26 to 61). Three patients reported having obtained a college degree. This group generated 42 facilitators and subsequently selected 13 as relatively more influential with respect to their individual medication decision-making processes (Fig. 3b; see Additional file 8 for more details). Six facilitators were each endorsed as influential by at least two patients. Together, the six facilitators were assigned 69 % of the weighted votes that were available for prioritizing perceived influence. These facilitators reflected (1) the desire to live (endorsed by 3 out of 7 patients; 21 % weighted votes), (2) concern for their dependents (endorsed by 3 out of 7 patients; 14 % weighted votes), (3) flare-up prevention (endorsed by 2 out of 7 patients; 12 % weighted votes), (4) medication affordability (endorsed by 2 out of 7 patients; 9 % weighted votes), (5) having short-duration treatment (endorsed by 2 out of 7 patients; 7 % weighted votes), and (6) medication with a minimum of side effects (endorsed by 2 out of 7 patients; 5 % weighted votes) (Fig. 3b; see Additional file 8 for more details).

## Discussion

This is the first detailed mixed methods study (qualitative and quantitative) of facilitators of decision-making related to medications for lupus nephritis. We purposefully oversampled African-American and Hispanic-American women since the medication adherence is lower and outcomes are worse compared with Caucasians with lupus [16, 17]. We identified several key facilitators to medication decision-making in our study, including effective patient-physician communication regarding benefits/harms, patient desire to live a normal life and concern for their dependents, experiencing benefits including improved quality of life and symptom relief and few/infrequent/no harms with lupus medications, and their affordability. Our study differs from the previous work in this area in three major respects: (1) we focused on facilitators to medication decision-making, not adherence to lupus medication, an important but different disease management construct; (2) we oversampled racial/ethnic minorities to increase the generalizability of study findings to the population of patients most severely affected by lupus nephritis; and (3) we used NGT, which allows both qualitative and quantitative assessment. In conjunction with our recently completed work on barriers to lupus nephritis medication decision-making [20], this new knowledge is the first step in the development of an effective patient decision aid.

All groups identified the benefit of effective communication with their health-care provider as a key facilitator to taking immunosuppressives regularly. Interestingly, in some groups, there was more than one concept mapping to this construct. This was an impressive finding since it indicates that patients value communication very highly in their decision to take or not to take lupus medications. The key concepts/domains facilitating patient decision-making related to lupus medications centered on the critical need to have better knowledge of the effectiveness of the medications. Patients with lupus emphasized including the following facilitators in effective patient-physician communication: medication benefits, how their medication does or does not impact other conditions, the positive impact of medication on their quality of life, how medication helps  to slow down the progression of disease and allows independence, avoids hospital admissions, and avoids too many doctor visits and dialysis and how the medication leads to clear benefits to them. A recent systematic review of qualitative studies identified effective communication, preserving health, negotiating medication regimens, and financial burden among top themes for medication adherence in patients with lupus [13]. However, most studies included in the systematic review did not include racial minorities and focused on adherence rather than the decision to start lupus treatments including immunosuppressive therapy. Our study examined these facilitators in detail, included minority patients, and extended these findings to medication decision-making. Our findings differ a bit from a UK study that included 13 black patients and reported that the facilitators of regular intake of lupus medications were the belief that there was no effective therapeutic alternative to their prescribed medications and feelings of moral obligation or responsibility to others and the fear of worsening disease [9]. We found far more positive facilitators than reported in the previous study. Differences in health-care systems (payer versus socialized) and country setting (US versus UK) might explain differences in findings.

A majority of the groups also identified that drug side effects influenced their decision regarding lupus mediations. Important factors included not only a good knowledge and understanding of possible side effects but also patient experience that side effects were minimal. Thus, both the knowledge of potential side effects and a personal experience of few or no side effects with lupus medications were important facilitators to decision-making about lupus treatments. While it is intuitive to think of fear of side effects as barriers to medication decision-making in lupus and other chronic conditions, our study shows that an adequate knowledge or experience (or both) can be a facilitator in this process.

Another important motivating factor for patients was symptom relief they experienced with lupus medications. This finding aligns with a similar finding in the systematic review that preserving health was a facilitator to medication adherence in lupus [13] and extends it to lupus medication decision-making. Similarly, medication adherence in other chronic conditions is better with medications that are associated with symptom relief [21] or when the disease is symptomatic or both [22, 23]. However, this facilitator will apply only to patients already taking other lupus medications or at the time of retreatment with the same or similar medications.

Patients recognized the importance of lupus medications in prolonging life as well as improving their quality of life and getting back to a normal way of living. As a chronic autoimmune condition, lupus is associated with significant morbidity and mortality in young patients in their productive years. We found  that the recognition of illness severity and the reversal of disease impact on life by lupus medications were perceived as facilitators and helped patients make a decision favoring lupus medications.

Medication affordability was another facilitator for lupus medication decision-making. In general, patients report medication cost to be barrier to their use of medications. Most medications for lupus are generic and usually are covered by most insurance plans or provided as part of patient assistance programs. We have included this message regarding cost of medications in our lupus patient decision aid, which is currently being tested in a randomized trial. In one of the nominal groups, one patient endorsed “The fewer the medicine, the better” as a facilitator for medication decision-making. This is not an uncommon struggle many patients have with poly-pharmacy and highlights the impact of poly-pharmacy on medication decision-making and adherence. This topic is an important consideration for discussion at the time of giving a new prescription to the patient, especially for organ-saving medications used in the treatment of lupus.

In a previous study, patients with lupus identified a lack of understanding of their disease, lack of understanding of psychosocial impact of lupus and their needs by their lupus health-care experts, and lack of information to address these needs [4]. Minority patients with lupus (n = 29) identified the desire for lupus education, need for assistance navigating the healthcare system, isolation at the time of diagnosis, and the emotional and physical barriers to care as the top targets; most (69 %) favored a peer support intervention [3].

Some limitations must be considered while interpreting these study findings. Since our NGTs were performed in females, findings may not be generalizable to men with lupus. It is possible that facilitators differ by gender, and this should be explored in future studies. Future studies should also consider whether lupus medication decision-making processes differ by the route of medication administration (intravenous versus oral) and by the type of lupus medication (immunosuppressives versus non-immunosuppressives versus biologics). Our study does not address decision-making for other medications that patients with lupus take, such as anti-hypertensives, lipid-lowering mediations, and cardiac medications. Future research should address these important issues.

## Conclusions

This mixed methods study used NGT to assess the facilitators to the decision-making regarding medications for lupus nephritis in African-American, Hispanic, and Caucasian women with lupus. Several facilitators were identified and prioritized by women with lupus. Considering our study findings and previous research, we conclude that engaging patients actively to acquire needed information to facilitate medication decision-making is needed. Funded by the Patient-Centered Outcomes Research Institute, we have now developed a lupus decision aid based on the comprehensive knowledge of these facilitators as well as barriers to medication decision-making [20]. A randomized trial to assess whether this patient decision aid is effective in improving patient medication decision-making regarding lupus medications is under way. If found to be effective, this decision aid will be available in the public domain for use by lupus patients and their care providers. An effective lupus patient guide and decision aid that provides balanced scientific information about lupus medication from a patient perspective and in words patients can understand and relate to can make it easier for patients to make informed decisions about lupus medications.

## Additional files

Additional file 1:
**Prioritized facilitators to help patients make decisions about treatment choices in AA1 (n = 9) (UAB, Birmingham, AA, 7 low SES, 2 high SES).** This table provides a list of prioritized facilitators to help patients make decisions about treatment choices in African-American patients in nominal group 1. *AA* African-American, *SES* socioeconomic status, *UAB* University of Alabama at Birmingham (DOCX 14 kb) Additional file 2:
**Prioritized facilitators in AA2 (UAB, Birmingham, AA, 6 low SES, 1 high SES).** This table provides a list of prioritized facilitators to help patients make decisions about treatment choices in African-American patients in nominal group 2. *AA* African-American, *SES* socioeconomic status, *UAB* University of Alabama at Birmingham (DOC 43 kb) Additional file 3:
**Prioritized facilitators in AA3 (UAB, Birmingham, AA, 3 low SES, 4 high SES).** This table provides a list of prioritized facilitators to help patients make decisions about treatment choices in African-American patients in nominal group 3. *AA* African-American, *SES* socioeconomic status, *UAB* University of Alabama at Birmingham (DOC 42 kb) Additional file 4:
**Prioritized facilitators in AA4 (n = 4)** ^**a**^ **(UAB, Birmingham, AA, 2 low SES, 2 high SES).** This table provides a list of prioritized facilitators to help patients make decisions about treatment choices in African-American patients in nominal group 4. *AA* African-American, *SES* socioeconomic status, *UAB* University of Alabama at Birmingham (DOC 35 kb) Additional file 5:
**Prioritized facilitators in CA1 (n = 6) (UAB, Birmingham, CA, 5 low SES, 1 high SES).** This table provides a list of prioritized facilitators to help patients make decisions about treatment choices in Caucasian patients in nominal group 1. *CA* Caucasian American, *SES* socioeconomic status, *UAB* University of Alabama at Birmingham (DOC 39 kb) Additional file 6:
**Prioritized facilitators in CA2 (n = 6) (UAB, Birmingham, CA, 2 low SES, 4 high SES).** This table provides a list of prioritized facilitators to help patients make decisions about treatment choices in Caucasian patients in nominal group 2. *CA* Caucasian American, *SES* socioeconomic status, *UAB* University of Alabama at Birmingham (DOC 43 kb) Additional file 7:
**Prioritized facilitators in HA1 (n = 6) (UCSF, San Francisco, HA, 5 low SES, 1 high SES).** This table provides a list of prioritized facilitators to help patients make decisions about treatment choices in Hispanic patients in nominal group 1. *HA* Hispanic American, *SES* socioeconomic status, *UCSF* University of California at San Francisco (DOC 39 kb) Additional file 8:
**Prioritized facilitators in HA2 (n = 7) (UCSF, San Francisco, HA, 4 low SES, 3 high SES).** This table provides a list of prioritized facilitators to help patients make decisions about treatment choices in Hispanic patients in nominal group 2. *HA* Hispanic American, *SES* socioeconomic status, *UCSF* University of California at San Francisco (DOCX 14 kb)

## Abbreviations

NGT: Nominal group technique
SD: Standard deviation
UAB: University of Alabama at Birmingham
UCSF: University of California at San Francisco
