# Supplementary material for: Minorities with lupus nephritis and medications: a study of facilitators to medication decision-making
Source: Arthritis Res Ther. 2015 Dec 17;17:367. doi: 10.1186/s13075-015-0883-z (PMC4704543; doi:10.1186/s13075-015-0883-z)
Supplement: Additional file 1: — Prioritized facilitators to help patients make decisions about treatment choices in AA1 (n = 9) (UAB, Birmingham, AA, 7 low SES, 2 high SES). This table provides a list of prioritized facilitators to help patients make decisions about treatment choices in African-American patients in nominal group 1. AA African-American, SES socioeconomic status, UAB University of Alabama at Birmingham (DOCX 14 kb) [file 13075_2015_883_MOESM1_ESM.docx]

**ADDITIONAL FILE**

**Additional File 1. Prioritized Facilitators to helping patients make decision about treatment choices in AA1 (n=9)** (UAB, Birmingham, AA, 7 low SES, 2 high SES)

| Response # | Responses | # of Votes | Votes Assigned | Sum of Votes | Weighted  Votes (%) |
| --- | --- | --- | --- | --- | --- |
| 13 | My kids are the reason and I want be there for them (if I don’t take the medication, I am a mess) | 5 | 3,3,3,3,1 | 13 | 24.07 |
| 5 | My will to live a long life/longevity | 5 | 3,3,3,2,2 | 13 | 24.07 |
| 24 | To avoid being admitted to the hospital | 2 | 3,2 | 5 | 9.26 |
| 18 | Not having to have as many doctor visits | 3 | 1,1,1 | 3 | 5.56 |
| 3 | Symptoms suffered prior to being diagnosed | 1 | 3 | 3 | 5.56 |
| 1 | Understanding how important the medication is for me | 1 | 2 | 2 | 3.70 |
| 2 | If it does not have extreme side effects | 1 | 2 | 2 | 3.70 |
| 6 | Hoping to get better by taking medication | 1 | 2 | 2 | 3.70 |
| 9 | The comfort that medication provides | 1 | 2 | 2 | 3.70 |
| 15 | Knowing how long the medication has been used for treatment and how successful it is | 1 | 2 | 2 | 3.70 |
| 17 | Understanding the different health risks that came along/associated with taking the medication | 1 | 2 | 2 | 3.70 |
| 16 | To be able to regain my independence | 1 | 1 | 1 | 1.85 |
| 19 | Being able to do the stuff that my spouse expects me to do | 1 | 1 | 1 | 1.85 |
| 22 | To be able to avoid dialysis | 1 | 1 | 1 | 1.85 |
| 23 | Seeing how much research is available about the medication | 1 | 1 | 1 | 1.85 |
| 27 | To be able to share the benefit of taking the medication with others | 1 | 1 | 1 | 1.85 |
| Total |  | 27 |  | 54 | 100.00 |
