# Supplementary material for: Minorities with lupus nephritis and medications: a study of facilitators to medication decision-making
Source: Arthritis Res Ther. 2015 Dec 17;17:367. doi: 10.1186/s13075-015-0883-z (PMC4704543; doi:10.1186/s13075-015-0883-z)
Supplement: Additional file 2: — Prioritized facilitators in AA2 (UAB, Birmingham, AA, 6 low SES, 1 high SES). This table provides a list of prioritized facilitators to help patients make decisions about treatment choices in African-American patients in nominal group 2. AA African-American, SES socioeconomic status, UAB University of Alabama at Birmingham (DOC 43 kb) [file 13075_2015_883_MOESM2_ESM.doc]

**Additional File 2. Prioritized Facilitators in AA2** (UAB, Birmingham, AA, 6 low SES, 1 high SES)

| Response # | Responses | # of Votes | Votes Assigned | Sum of Votes | Weighted  Votes (%) |
| --- | --- | --- | --- | --- | --- |
| 4 | Knowing about the side effects | 2 | 3,3 | 6 | 14.29 |
| 23 | Knowing that it won't aggravate other conditions (e.g., having side effects on lung) | 2 | 3,3 | 6 | 14.29 |
| 5 | Getting more education about the treatment for this specific disease so that I can avoid medication overlap/duplication | 2 | 3,3 | 6 | 14.29 |
| 14 | To be able to live a normal life without so many complications | 2 | 3,2 | 5 | 11.90 |
| 11 | Being able to afford it | 3 | 1,1,1 | 3 | 7.14 |
| 1 | Knowing that medicine is going to help and not cause any other problems | 2 | 2,1 | 3 | 7.14 |
| 6 | Hearing about people who have been treated with this medication and that it worked | 1 | 2 | 2 | 4.76 |
| 7 | To protect the good cells of your body | 1 | 2 | 2 | 4.76 |
| 8 | If I will be able to stop taking medicine after a period of time | 1 | 2 | 2 | 4.76 |
| 16 | Would like more research on the failure of medication | 1 | 2 | 2 | 4.76 |
| 26 | Knowing the adverse effect of taking or not taking the medication--risks vs. benefits | 1 | 2 | 2 | 4.76 |
| 2 | The fewer the medicine, the better | 1 | 1 | 1 | 2.38 |
| 34 | If it does not cause mouth dryness & hair loss | 1 | 1 | 1 | 2.38 |
| 35 | It would be nice to know if the doctors are not being paid/sponsored for prescribing the medication | 1 | 1 | 1 | 2.38 |
| Total |  | 21 |  | 42 | 100.00 |
