# Supplementary material for: Minorities with lupus nephritis and medications: a study of facilitators to medication decision-making
Source: Arthritis Res Ther. 2015 Dec 17;17:367. doi: 10.1186/s13075-015-0883-z (PMC4704543; doi:10.1186/s13075-015-0883-z)
Supplement: Additional file 3: — Prioritized facilitators in AA3 (UAB, Birmingham, AA, 3 low SES, 4 high SES). This table provides a list of prioritized facilitators to help patients make decisions about treatment choices in African-American patients in nominal group 3. AA African-American, SES socioeconomic status, UAB University of Alabama at Birmingham (DOC 42 kb) [file 13075_2015_883_MOESM3_ESM.doc]

**Additional File 3. Prioritized Facilitators in AA3 (UAB, Birmingham, AA, 3 low SES, 4 high SES)**

| Response # | Responses | # of Votes | Votes Assigned | Sum of Votes | Weighted  Votes (%) |
| --- | --- | --- | --- | --- | --- |
| 10 | If it is affordable | 4 | 2,2,1,1 | 6 | 14.29 |
| 3 | Getting relief for some or all my symptoms | 2 | 3,3 | 6 | 14.29 |
| 18 | Knowing how effective the medication is | 2 | 3,3 | 6 | 14.29 |
| 30 | Knowing if it is approved by FDA or is it just an experimental medication | 2 | 3,2 | 5 | 11.90 |
| 1 | Knowing what the side effects are | 2 | 2,2 | 4 | 9.52 |
| 12 | Knowing what the benefits are for me if I take the medication | 2 | 3,1 | 4 | 9.52 |
| 17 | Having a great support system | 1 | 3 | 3 | 7.14 |
| 2 | Wanting to be healthier to work/to live | 1 | 2 | 2 | 4.76 |
| 21 | Knowing how it affects other patients with the same symptoms | 1 | 2 | 2 | 4.76 |
| 7 | Hearing about studies that have been done and if they were successful | 1 | 1 | 1 | 2.38 |
| 9 | Knowing that the medication won't affect my ability to have children | 1 | 1 | 1 | 2.38 |
| 32 | Knowing that this medication won't interfere my daily activities | 1 | 1 | 1 | 2.38 |
| 37 | Having trust in your health provider | 1 | 1 | 1 | 2.38 |
| Total |  | 21 |  | 42 | 100.00 |
