# Supplementary material for: Minorities with lupus nephritis and medications: a study of facilitators to medication decision-making
Source: Arthritis Res Ther. 2015 Dec 17;17:367. doi: 10.1186/s13075-015-0883-z (PMC4704543; doi:10.1186/s13075-015-0883-z)
Supplement: Additional file 4: — Prioritized facilitators in AA4 (n = 4) a (UAB, Birmingham, AA, 2 low SES, 2 high SES). This table provides a list of prioritized facilitators to help patients make decisions about treatment choices in African-American patients in nominal group 4. AA African-American, SES socioeconomic status, UAB University of Alabama at Birmingham (DOC 35 kb) [file 13075_2015_883_MOESM4_ESM.doc]

**Additional File 4. Prioritized Facilitators in AA4 (n=4)**^a^ (UAB, Birmingham, AA, 2 low SES, 2 high SES)

| Response # | Responses | # of Votes | Votes Assigned | Sum of Votes | Weighted  Votes (%) |
| --- | --- | --- | --- | --- | --- |
| 1 | Wanting to live as normal life as possible | 2 | 3,3 | 6 | 25.00 |
| 15 | Low cost | 3 | 3,1,1 | 5 | 20.83 |
| 2 | Not having side effects or do not have to worry about side effect | 2 | 2,1 | 3 | 12.50 |
| 5 | Seeing decreased symptoms of the disease when taking the medication | 1 | 3 | 3 | 12.50 |
| 4 | Having less risk factor or less possibilities of medication cause cancer etc. | 1 | 2 | 2 | 8.33 |
| 6 | Having proof that the medication works | 1 | 2 | 2 | 8.33 |
| 22 | Other patients' outcome have improved for whom have taken the prescribed medication by doctors | 1 | 2 | 2 | 8.33 |
| 23 | Knowing the medicine is helping | 1 | 1 | 1 | 4.17 |
| Total |  | 12 |  | 24 | 100.00 |

Note: ^a^ Caution should be used when using the results given the small n because it does not meet the recommended number.
