# Supplementary material for: Minorities with lupus nephritis and medications: a study of facilitators to medication decision-making
Source: Arthritis Res Ther. 2015 Dec 17;17:367. doi: 10.1186/s13075-015-0883-z (PMC4704543; doi:10.1186/s13075-015-0883-z)
Supplement: Additional file 5: — Prioritized facilitators in CA1 (n = 6) (UAB, Birmingham, CA, 5 low SES, 1 high SES). This table provides a list of prioritized facilitators to help patients make decisions about treatment choices in Caucasian patients in nominal group 1. CA Caucasian American, SES socioeconomic status, UAB University of Alabama at Birmingham (DOC 39 kb) [file 13075_2015_883_MOESM5_ESM.doc]

**Additional File 5. Prioritized Facilitators in CA1 (n=6)** (UAB, Birmingham, CA, 5 low SES, 1 high SES)

| Response # | Responses | # of Votes | Votes Assigned | Sum of Votes | Weighted  Votes (%) |
| --- | --- | --- | --- | --- | --- |
| 5 | Because the doctors know more than I do | 3 | 3,3,1 | 7 | 19.44 |
| 1 | If the side effects are not significant | 3 | 2,2,1 | 5 | 13.89 |
| 9 | The desire to feel better | 2 | 3,2 | 5 | 13.89 |
| 31 | Desire to stay active/healthy | 2 | 3,1 | 4 | 11.11 |
| 16 | Thinking that it will extend your life span | 2 | 2,1 | 3 | 8.33 |
| 4 | Hoping that it will slow down the progression of disease | 1 | 3 | 3 | 8.33 |
| 10 | Seeing an improvement in overall health | 1 | 3 | 3 | 8.33 |
| 2 | Researching on my own | 1 | 2 | 2 | 5.56 |
| 18 | Desire to be with my family--my children | 1 | 2 | 2 | 5.56 |
| 11 | Not wanting to see organ shut down | 1 | 1 | 1 | 2.78 |
| 14 | My present health status/state and knowledge of my own body | 1 | 1 | 1 | 2.78 |
| Total |  | 18 |  | 36 | 100.00 |
