# Supplementary material for: Minorities with lupus nephritis and medications: a study of facilitators to medication decision-making
Source: Arthritis Res Ther. 2015 Dec 17;17:367. doi: 10.1186/s13075-015-0883-z (PMC4704543; doi:10.1186/s13075-015-0883-z)
Supplement: Additional file 6: — Prioritized facilitators in CA2 (n = 6) (UAB, Birmingham, CA, 2 low SES, 4 high SES). This table provides a list of prioritized facilitators to help patients make decisions about treatment choices in Caucasian patients in nominal group 2. CA Caucasian American, SES socioeconomic status, UAB University of Alabama at Birmingham (DOC 43 kb) [file 13075_2015_883_MOESM6_ESM.doc]

**Additional File 6. Prioritized Facilitators in CA2 (n=6)** (UAB, Birmingham, CA, 2 low SES, 4 high SES)

| Response # | Responses | # of Votes | Votes Assigned | Sum of Votes | Weighted  Votes (%) |
| --- | --- | --- | --- | --- | --- |
| 6 | Having good rapport with doctors and trusting them | 2 | 3,2 | 5 | 13.89 |
| 13 | Understanding what am I taking | 2 | 2,1 | 3 | 8.33 |
| 1 | Limited side effects | 2 | 2,1 | 3 | 8.33 |
| 2 | Having resources to pay for the medications | 1 | 3 | 3 | 8.33 |
| 3 | If I knew if and when I would feel better | 1 | 3 | 3 | 8.33 |
| 4 | Having proof of concept (evidence/statistics--that the medication works) | 1 | 3 | 3 | 8.33 |
| 11 | Doctor's knowledge of the medications prescribed | 1 | 3 | 3 | 8.33 |
| 38 | Knowing if taking the medication will improve your quality of life (QoL) | 1 | 3 | 3 | 8.33 |
| 22 | Having a positive outlook on my diagnosis and treatment | 2 | 1,1 | 2 | 5.56 |
| 5 | Just knowing that your doctors are working together for your treatment | 1 | 2 | 2 | 5.56 |
| 10 | Having a strong support systems (family encourage--want you to get better) | 1 | 2 | 2 | 5.56 |
| 27 | If the doctor tell you what he wants and expects to see happen with you if you take the medication | 1 | 2 | 2 | 5.56 |
| 9 | Understanding the pros and cons of taking vs. not taking medications | 1 | 1 | 1 | 2.78 |
| 15 | If the medications did not make you gain weight | 1 | 1 | 1 | 2.78 |
| Total |  | 18 |  | **36** | **100.00** |
