# Supplementary material for: Minorities with lupus nephritis and medications: a study of facilitators to medication decision-making
Source: Arthritis Res Ther. 2015 Dec 17;17:367. doi: 10.1186/s13075-015-0883-z (PMC4704543; doi:10.1186/s13075-015-0883-z)
Supplement: Additional file 7: — Prioritized facilitators in HA1 (n = 6) (UCSF, San Francisco, HA, 5 low SES, 1 high SES). This table provides a list of prioritized facilitators to help patients make decisions about treatment choices in Hispanic patients in nominal group 1. HA Hispanic American, SES socioeconomic status, UCSF University of California at San Francisco (DOC 39 kb) [file 13075_2015_883_MOESM7_ESM.doc]

**Additional File 7. Prioritized Facilitators in HA1 (n=6)** (UCSF, San Francisco, HA, 5 low SES, 1 high SES)

| Response # | Responses | # of Votes | Votes Assigned | Sum of Votes | Weighted  Votes (%) |
| --- | --- | --- | --- | --- | --- |
| 31 | To be able to get back to your normal life | 3 | 3, 1,1 | 5 | 13.89 |
| 6 | Getting an explanation of side effects and the benefits the medicine has for my kidney | 2 | 2,2 | 4 | 11.11 |
| 1 | If it gives good results-outcome or a cure | 2 | 2,1 | 3 | 8.33 |
| 15 | To stop feeling weak and tired | 2 | 2,1 | 3 | 8.33 |
| 25 | To be able to live longer | 1 | 3 | 3 | 8.33 |
| 23 | To be able to have a healthy pregnancy in the future | 1 | 3 | 3 | 8.33 |
| 9 | To stop the swelling | 1 | 3 | 3 | 8.33 |
| 5 | Worrying about the possible risk if the medicine is not taken | 1 | 3 | 3 | 8.33 |
| 35 | Knowing how to choose the night foods to take with medicine | 1 | 3 | 3 | 8.33 |
| 14 | To stop joint pain | 1 | 2 | 2 | 5.56 |
| 36 | You've tried everything else and nothing has worked | 1 | 2 | 2 | 5.56 |
| 12 | Having some education about the medications and treatments | 1 | 1 | 1 | 2.78 |
| 37 | Getting education about how the medicine would affect my pregnancy | 1 | 1 | 1 | 2.78 |
| Total |  | 18 |  | 36 | 100.00 |
