# Supplementary material for: Minorities with lupus nephritis and medications: a study of facilitators to medication decision-making
Source: Arthritis Res Ther. 2015 Dec 17;17:367. doi: 10.1186/s13075-015-0883-z (PMC4704543; doi:10.1186/s13075-015-0883-z)
Supplement: Additional file 8: — Prioritized facilitators in HA2 (n = 7) (UCSF, San Francisco, HA, 4 low SES, 3 high SES). This table provides a list of prioritized facilitators to help patients make decisions about treatment choices in Hispanic patients in nominal group 2. HA Hispanic American, SES socioeconomic status, UCSF University of California at San Francisco (DOCX 14 kb) [file 13075_2015_883_MOESM8_ESM.docx]

**Additional File 8. Prioritized Facilitators in HA2 (n=7)** (UCSF, San Francisco, HA, 4 low SES, 3 high SES)

| Response # | Responses | # of Votes | Votes Assigned | Sum of Votes | Weighted  Votes (%) |
| --- | --- | --- | --- | --- | --- |
| 19 | Wanting to live | 3 | 3,3,3 | 9 | 21.43 |
| 11 | Thinking about the people who depend on me | 3 | 2,2,2 | 6 | 14.29 |
| 16 | To prevent a flare-up | 2 | 3,2 | 5 | 11.90 |
| 9 | Not costing more than I can afford | 2 | 3,1 | 4 | 9.52 |
| 8 | Knowing how long the medicine has to be taken--the shorter the better | 2 | 2,1 | 3 | 7.14 |
| 3 | Having some patient education about the medication as opposed to the doctor saying "just take it" | 1 | 3 | 3 | 7.14 |
| 15 | Being informed about the specific side effects that I can expect from taking the medication | 1 | 3 | 3 | 7.14 |
| 24 | Knowing how long it will be before the medicine takes effect? | 1 | 2 | 2 | 4.76 |
| 2 | If the medicine had the least amount of side effects | 2 | 1,1 | 2 | 4.76 |
| 41 | Understanding how the medicine will affect my ability to have children in the future | 1 | 2 | 2 | 4.76 |
| 4 | I don't want to have dialysis again | 1 | 1 | 1 | 2.38 |
| 7 | I don't want to get worse | 1 | 1 | 1 | 2.38 |
| 32 | Having some type of plan to get off of the medicine | 1 | 1 | 1 | 2.38 |
| Total |  | 21 |  | 42 | 100.00 |
